# Mental disorder and PTSD in Syria during wartime: a nationwide crisis

**DOI:** 10.1186/s12888-020-03002-3

**Published:** 2021-01-02

**Authors:** Ameer Kakaje, Ragheed Al Zohbi, Osama Hosam Aldeen, Leen Makki, Ayham Alyousbashi, Mhd Bahaa Aldin Alhaffar

**Affiliations:** 1 Faculty of Medicine, Damascus University, 31037 Damascus, Syria; 2 Faculty of Medicine, Aleppo University, Aleppo, Syria; 3 Department of Experimental Surgery, McGill University, Quebec, Canada; 4 Department of Periodontology, Faculty of Dentistry, Damascus University, Alkhateeb sq, Damascus, Syria

**Keywords:** Conflict, Global health, K10, Mental distress, PTSD, Public health, Refugees, Social support, Syria, War

## Abstract

**Background:**

Syria has experienced war since 2011, leaving over 80% under the poverty line and millions displaced. War and its retaliations have significantly impacted the mental health of Syrians. This study evaluates the post-traumatic stress disorder (PTSD), and the severity of the mental distress caused by war and other factors such as low social support. This study also evaluates other variables and compares the findings with those of multiple studies on Syria and refugees.

**Methods:**

This is a cross-sectional study that included people who lived in Syria in different governorates. Online surveys were distributed into multiple online groups and included the Kessler 10 (K10) scale which screens for anxiety and depression, the Screen for Posttraumatic Stress Symptoms (SPTSS) tool, the Multidimensional Scale of Perceived Social Support, and questionnaires on demographic and war-related factors.

**Results:**

Our study included 1951 participants, of which, 527 (27.0%) were males and 1538 (78.8%) between the age of 19 and 25. Among participants, 44% had likely severe mental disorder, 27% had both likely severe mental disorder and full PTSD symptoms, 36.9% had full PTSD symptoms, and only 10.8% had neither positive PTSD symptoms nor mental disorder on the K10 scale. Around 23% had low overall support. Half of the responders were internally displaced, and 27.6% were forced to change places of living three times or more due to war. Around 86.6% of the responders believed that the war was the main reason for their mental distress. Those with high SPTSS and K10 scores were found to take more days off from work or school due to negative feelings and having somatic symptoms. Moreover, the number of times changing places of living due to war, educational level, and being distressed by war noise were the most prominent factors for more severe PTSD and mental distress. No differences in PTSD and mental disorder prevalence were noted in participants living in different governorates or among different types of jobs. A strong significant correlation (*r* = 0.623) was found between SPTSS and K10 scores.

**Conclusion:**

The conflict in Syria has left the population at great risk for mental distress which was higher compared to Syrian refugees elsewhere. Many measures with an emphasis on mental health are needed to help the people against a long-term avoidable suffering.

## Background

Warfare has acute and chronic effects on the community and is considered one of the most mentally stressful events that can be experienced by someone. The indirect effects of warfare can be disastrous as they can continue for years after the settling of the conflict. The aftermath of war is challenging, from shortage of resources such as food, water, fuel and medical equioment, to displacement and disease outbreak [1]. Since the beginning of the Syrian conflict in 2011, many people have been wounded, 511,000 have died [2], more than 5.6 million have been externally displaced [3], and over 6.2 million have been internally displaced [4]. In 2014, it was estimated that 82.5% of Syrians lived below the poverty line. Syria’s deteriorating economy presents a challenge in terms of resource allocation to the health sector as only 1.5 hospital beds and 1.22 physicians were available for every1000 people, making mental medical conditions almost impossible to treat [5]. Moreover, the economy and health sector are much worse at the time of this study.

The damage to the medical infrastructure and the loss of medical staff have greatly affected the health system in Syria. Mental health in Syria has been neglected for decades, with no efforts to reduce the stigma on mental health, and few psychologist and psychiatrist available. This has caused the mental health sector to suffer greatly compared with other medical specialties during the war. The education sector has also been significantly injured after many facilities being destroyed by the conflict with the destruction of the Syrian social structure, most children had to grow up in a war-burdened environment that scarred their normal development [6]. These disturbances and loss of both social and emotional support have led to patterns of severe mental trauma [7] which left many people paralysed and incapable of facing everyday challenges, mainly due to years, if not decades, of severe mental distress and high financial burdens threatening several generations. Furthermore, the post-traumatic effects will have a longer-lasting impact which may continue for several years after the conflict resolves [8–10].

Many Syrians have fled the country and sought asylum in different countries. Although most of them lived in a relatively safer environment after facing grave dangers to reach their destination, they suffered mentally as they were far from their homeland, family members, friends, and usual work environment, all while experiencing bad living conditions in case they were living in camps. People who left the conflict zone have also developed mental disorders that could be different from those who have stayed [11]. Furthermore, several studies indicate that mental distress is higher in conflict areas. This study evaluates mental disorders, post-traumatic stress disorder (PTSD), and the amount of social support among Syrians who remained in Syria as well as the relvevant factors affecting the mental health in war time. This study used self-reported questionnaires due to the lack of funding allocated to medical research in Syria and unavailable resources for proper diagnosis [12–16]. This study also assessed other variables such as demographics and those which are relateed to war and compared them with other studies for a better evaluation of the mental disorders among Syrians and refugees.

## Methods

### Sampling

This is a cross-sectional study conducted in Syria from 3/3/2019 to 24/3/2019. Online surveys in Arabic were distributed to participants from several Syrian governorates. Only participants who lived in Syria at the time of the survey and answered the demographic and K10 questions were enrolled. The questionnaires were posted online twice each day at 10 AM and 10 PM in social media groups with different topics such as educational, cuisine, market, entertainment, cultural, musical etc. Any participant who spoke Arabic, lived in Syria at the time of the study, and answered the required questions was included.

### Consent and approval for study

The informed consent was taken online for using and publishing the data before carrying on with the survey. Confidentiality was assured and no identity-revealing questions were asked. This method was approved by Damascus University.

Our study protocol and ethical aspects were reviewed and approved by Damascus University deanship in Damascus, Syria.

### Questionnaires

#### Socioeconomic status (SES)

There is no suitable validated SES measurement to use in the Syrian population as we cannot ask directly about the monthly salary due to cultural norms that make it inappropriate. In addition, there are huge differences between the normal wage in Syria and other countries. Therefore, SES was assessed through three criteria; 1) the level of education, 2) the profession of the participant or that of the working family member/partner, 3) the monthly income adequacy of the individual who is the main source of income in the household. SES was then divided into four different categories: lower, upper-lower, middle, and upper.

#### Screening for mental disorder

Kessler 10 is typically used in screening surveys to measure mental distress [17–19]. An Arabic validate version of the K10 + LM questionnaires was used [17, 18]. It is a self-reported measure that assesses anxiety and depression symptoms over the previous 4 weeks. It contains ten questions with scores ranging from 10 to 50. Subjects who score under 20 are likely to be well, those who score 20–24 could have a mild mental disorder, those who score 25–29 have moderate mental disorder, and finally those who score 30 and above have a severe mental disorder. Each question has five possible responses with scores ranging from 1 to 5.

#### Social support

An Arabic version of the Multidimensional Scale of Perceived Social Support (MSPSS) was used to measure the social support that the individual receives from their social network, including friends, family, and the significant other [20, 21]. The measure contained 12 questions with four questions for each source. Support received was then divided into three categories: low support with a mean score of 1 to 2.9, moderate support with a mean score of 3 to 5, and finally high support with a mean score of 5.1 to 7. The scores and measures were validated in Arabic [21]. The questionnaire and its translation are available [22].

#### PTSD

The Screen for Posttraumatic Stress Symptoms (SPTSS) tool was used; a self-reporting screening method for PTSD that is very concise, suitable, easy to understand and does not focus on specific traumatic events. SPTSS is not meant to be a definitive diagnostic tool for PTSD. It measures three PTSD clusters over the previous 4 weeks; avoidance with 7 items, hyper-hyper-arousal with 5 items, and re-experience with 5 items. Each question has 5 potential responses, ranging from “Not at all” to “More than once every day”, with scores ranging from 0 to 4. However, when calculating the final score for each cluster, the first two scores represent 0 and the other three scores represent 1. Scoring 3 or more on avoidance cluster, 2 or more on re-experience, and 1 or more on excessive hyper-arousal is an indication of PTSD for that specific cluster. The scores and measures are validated in Arabic [22, 23]. This scale, however, is based on the diagnostic and statistical manual of mental disorders (DSM) IV. These three clusters are close to what is used in the International Classification of Disease 11 (ICD-11) criteria although using DSM IV criteria usually identifies cases with less severe trauma exposure [24].

#### Other questions

Subjects were asked basic demographic questions such as gender, age, educational level, governorate of current living, and whether they had consanguineous parents. Subjects were also asked a few questions, both directly and indirectly, about war, including changing place of living due to war, losing someone close, and being distressed from war noises. Finally, subjects were asked if they had any chronic medical condition.

This study defined third-degree consanguineous parents as having parents who were first cousins, and fourth-degree consanguineous parents as having parents who were second cousins, or second cousins once removed who is either a child of one’s second cousin, or one’s parent’s second cousin.

### Data process

Data were processed using IBM SPSS software version 26 for Windows (SPSS Inc., IL, USA). Chi-square was used with nominal variables such as in (Table 1) while one-way analysis of variance (ANOVA), and independent t-tests were performed to determine statistical significance when having a numeric variable with a nominal one such as in (Tables 2 and 3). Pearson correlation was also used when having two numeric variables.

**Table 1:** Comparing Each SPTSS Cluster with Other Variables

	Avoidance					Arousal					Re-experience					
	Negative(*n* = 910) - %		Positive (*n* = 1040) - %		*P* value	Negative (*n* = 693) - %		Positive (*n* = 1257) - %		*P* value	Negative (*n* = 800) - %		Positive (*n* = 1150) - %		*P* value	*P* value^#^
**Gender**																
Male	247	27.1%	280	26.9%	NS	208	30.0%	319	25.4%	0.073 ^NS^	239	29.9%	288	25.0%	0.051 ^NS^	NS
Female	663	72.9%	760	73.1%		485	70.0%	938	74.6%		561	70.1%	862	75.0%		
**Consanguinity**																
No	656	72.1%	759	73.0%	NS	496	71.6%	919	73.1%	NS	591	73.9%	824	71.7%	NS	NS
Third-degree relatives	134	14.7%	166	16.0%		103	14.9%	197	15.7%		111	13.9%	189	16.4%		
Fourth-degree relatives	53	5.8%	44	4.2%		42	6.1%	55	4.4%		47	5.9%	50	4.3%		
Distant relatives	67	7.4%	71	6.8%		52	7.5%	86	6.8%		51	6.4%	87	7.6%		
**Marital status**																
Single	750	82.5%	866	83.4%	NS	574	82.9%	1042	83.0%	NS	661	82.7%	955	83.2%	NS	NS
Engaged	47	5.2%	39	3.8%		31	4.5%	55	4.4%		31	3.9%	55	4.8%		
Married	97	10.7%	120	11.6%		78	11.3%	139	11.1%		94	11.8%	123	10.7%		
Divorced	10	1.1%	11	1.1%		6	0.9%	15	1.2%		9	1.1%	12	1.0%		
Widowed	5	0.6%	2	0.2%		3	0.4%	4	0.3%		4	0.5%	3	0.3%		
**Educational level**																
Primary School	2	0.2%	23	2.3%	< 0.0001	2	0.3%	25	1.9%	< 0.0001	5	0.6%	22	1.8%	0.004	0.001
High School	45	4.9%	81	7.8%		30	4.3%	96	7.6%		44	5.5%	82	7.1%		
University or any high institute	807	88.7%	864	83.2%		624	90.0%	1047	83.4%		700	87.5%	971	84.5%		
Masters or PhD	56	6.2%	70	6.7%		37	5.3%	89	7.1%		51	6.4%	75	6.5%		
**SES**																
Lower	14	1.5%	16	1.5%		8	1.2%	22	1.8%		9	1.1%	21	1.8%		
Upper Lower	199	21.9%	283	27.2%	0.028 ^NS^	136	19.6%	346	27.5%	0.003	176	22.0%	306	26.6%	0.055 ^NS^	0.005 ^NS^
Middle	490	53.8%	554	53.3%		393	56.7%	651	51.8%		435	54.4%	609	53.0%		
Upper	207	22.7%	187	18.0%		156	22.5%	238	18.9%		180	22.5%	214	18.6%		
**Working**																
No	497	65.0%	600	67.6%	NS	385	67.2%	712	66.0%	NS	433	64.5%	664	67.8%	NS	NS
Yes	267	35.0%	287	32.4%		188	32.8%	366	34.0%		238	35.5%	316	32.2%		
**Age groups**																
14–18	24	2.6%	27	2.6%		15	2.2%	36	2.9%		14	1.8%	37	3.2%		
19–25	720	79.1%	818	78.7%	< 0.0001	563	81.2%	975	77.6%	< 0.0001	639	79.9%	899	78.2%	< 0.0001	0.066 ^NS^
26–45	147	16.2%	186	17.9%		102	14.7%	231	18.4%		128	16.0%	205	17.8%		
46–65	19	2.1%	9	0.9%		13	1.9%	15	1.2%		19	2.4%	9	0.8%		
**Losing someone close due to war**																
No	351	38.6%	346	33.3%	0.039 ^NS^	270	39.0%	427	34.0%	0.067 ^NS^	339	42.4%	358	31.1%	< 0.0001	0.001
Yes	559	61.4%	694	66.7%		423	61.0%	830	66.0%		461	57.6%	792	68.9%		
**A relative being endangered by the war**																
No	138	15.2%	146	14.0%	NS	113	16.3%	171	13.6%	NS	132	16.5%	152	13.2%	NS	NS
Yes	772	84.8%	894	86.0%		580	83.7%	1086	86.4%		668	83.5%	998	86.8%		
**Distressed from war noises**																
Negative	195	21.4%	211	20.3%	NS	185	26.7%	211	17.6%	< 0.0001	196	24.5%	210	18.3%	0.001	0.003
Positive	715	78.6%	829	79.7%		508	73.3%	1036	82.4%		604	75.5%	940	81.7%		
**Changing place of living due to war**																
No	495	54.4%	482	46.3%		361	52.1%	616	49.0%		413	51.6%	564	49.0%		
Within the same city	195	21.4%	233	22.4%	0.004	138	19.9%	290	23.1%	NS	173	21.6%	255	22.2%	NS	0.010 ^NS^
With changing city	195	21.4%	293	28.2%		172	24.8%	316	25.1%		191	23.9%	297	25.8%		
Both	25	2.7%	32	3.1%		22	3.2%	35	2.8%		23	2.9%	34	3.0%		
**Number of times changing places of living due to war**																
Never	495	37.8%	482	31.5%		361	38.0%	616	32.5%		413	36.4%	564	33.1%		
Once	123	22.7%	101	16.7%	< 0.0001	103	23.8%	121	17.2%	0.001	107	22.4%	117	17.6%	0.016 ^NS^	< 0.0001
Twice	87	13.5%	124	15.9%		63	11.7%	148	16.5%		91	14.8%	120	14.8%		
Thrice and more	205	25.9%	333	35.9%		166	26.6%	372	33.8%		189	26.5%	349	34.5%		
**The main reason declared of stress in the last period:**^**a**^																
Educational	197	39.5%	191	31.0%		155	43.9%	233	30.6%		173	41.2%	215	30.9%		
Economical	58	11.6%	69	11.2%	0.014 ^NS^	39	11.0%	88	11.5%	0.0001	54	12.9%	73	10.5%	0.001	0.001
Social	212	42.5%	292	47.4%		143	40.5%	361	47.4%		164	39.0%	340	48.9%		
War-related	20	4.0%	30	4.9%		8	2.3%	42	5.5%		17	4.0%	33	4.7%		
Medical	8	1.6%	25	4.1%		5	1.4%	28	3.7%		11	2.6%	22	3.2%		
Other	4	0.8%	9	1.5%		3	0.8%	10	1.3%		1	0.2%	12	1.7%		
**Do you consider that the crisis was the main cause of your distress lately?**^**a**^																
No	155	22.4%	107	12.6%		133	25.6%	129	12.6%		127	20.9%	135	14.5%		
Kind of	326	47.2%	336	39.6%	< 0.0001	150	45.5%	426	41.7%	< 0.0001	294	48.4%	368	39.4%	< 0.0001	< 0.0001
Yes totally	210	30.4%	210	47.8%		319	28.9%	466	45.6%		186	30.6%	430	46.1%		

Where *NS* Not significant

Chi-square test was used in this table

^#^This *p* value is calculated between having no, one, two or three positive clusters

^a^These were not included in the regression as they do not generate extra results and they overlap with most variables

When using Bonferroni correction, *P* = (0.05\14) ≈ 0.004 or less to be statistically significant

**Table 2:** Demonstrate rrelationship between variables and K10 and mspss scores

Characteristic	K10 score				MSPSS Score			
	Mean	SD	***P*** value	F	Mean	SD	***P*** value	F
**Gender**								
Female	29.3	9.8	< 0.001	21.8	51.3	18.2	*NS*	0.9
Male	27.0	9.7			50.5	18.4		
**Consanguinity**								
No	28.8	9.9			51.0	18.4		
Yes third-degree relatives	28.7	9.5	0.038 ^*NS*^	2.8	50.5	18.6	*NS*	1.9
Yes fourth-degree relatives	26.1	9.4			55.4	16.9		
Yes but not close relatives	29.7	9.9			50.6	16.4		
**Marital status**								
Single	28.7	9.8	*NS*	0.2	50.7	18.2	0.002	4.4
Engaged	28.0	10.1			58.5	15.7		
Married	28.7	10.1			52.1	19.0		
Widowed	28.9	9.6			51.6	16.5		
Divorced	29.8	11.6			45.8	17.9		
**Educational level**								
Primary School	39.7	9.3			39.5	19.9		
High School	30.5	8.9	< 0.001	13	50.2	18.4	0.011 ^*NS*^	3.7
University or any high institute	28.4	9.8			51.3	18.1		
Masters or PhD	29.1	9.7			51.6	18.9		
**SES**								
Lower	32.0	11.1			38.2	17.8		
Upper Lower	30.4	9.8	< 0.001	8.5	48.0	18.1	< 0.001	15.9
Middle	28.3	9.7			51.4	18.3		
Upper	27.4	10.0			55.0	17.1		
**Working**								
No	28.9	9.8	*NS*	1.0	51.1	18.0	*NS*	0.1
Yes	28.4	9.8			51.3	18.5		
**Age groups**								
14–18	31.7	9.6			50.5	17.0		
19–25	28.6	9.8	< 0.001	8.1	51.2	18.1	*NS*	0.3
26–45	29.3	10.1			50.7	18.7		
46–65	20.8	6.7			48.5	22.2		
**Losing someone close due to war**								
No	28.0	9.7	0.013 ^*NS*^	6.2	51.7	18.1	*NS*	1.3
Yes	29.1	9.9			50.7	18.3		
**A relative being endangered by the war**								
No	28.1	9.6	*NS*	1.4	50.2	18.4	*NS*	0.7
Yes	28.8	9.9			51.2	18.2		
**Distressed from war noises**								
Negative	27.1	9.9	< 0.001	13.5	50.2	18.5	*NS*	1.3
Positive	29.1	9.8			51.3	18.2		
**Changing place of living due to war**								
No	28.0	9.6			52.2	18.3		
Within the same city	29.6	9.8	0.005 ^*NS*^	4.2	50.9	18.1	0.027 ^*NS*^	3.1
Changing the city	29.5	10.2			49.3	18.3		
Doing both	28.4	9.4			48.8	16.1		
**Number of times changing places of living due to war**								
No	28.0	9.6	< 0.001	8.8	52.2	18.3	0.011 ^*NS*^	3.7
Once	27.5	10.3			51.8	17.3		
Twice	29.3	9.6			50.6	17.8		
Thrice and more	30.4	9.9			49.0	18.5		
**The reason declared of stress in the last period:**^**a**^								
Educational			< 0.001	7.3	54.2	17.4	0.001	4.4
Economical	27.6	9.3			52.8	17.3		
Social	29.5	10.2			49.7	18.0		
War-related	30.4	10.2			49.9	19.8		
Medical	30.2	9.7			54.1	19.8		
Other	32.9	8.4			38.5	20.8		
**Do you consider that the crisis was the main cause of your distress lately?**^**a**^								
No	26.2	9.5	< 0.001	28.3	53.2	18.8	0.054^*NS*^	2.9
Kind of	28.5	9.9			51.2	18.1		
Yes totally	31.3	9.8			50.0	18.0		

One-way ANOVA test was used in this table

^a^These were not included in the regression as they do not generate extra results and they overlap with most variables

When using Bonferroni correction, *P* = (0.05\14) ≈ 0.004 or less to be statistically significant

**Table 3:** Comparing Each Support in MSPSS with K10 and SPTSS clusters

	Family Support			Friends support			Significant Other Support		
	Mean	SD	***P*** value	Mean	SD	***P*** value	Mean	SD	***P*** value
K 10									
No	21.0	6.6	< 0.001	17.2	7.0	< 0.001	19.6	7.3	< 0.001
Low	19.0	6.8		15.1	7.3		17.9	7.6	
Medium	18.3	7.2		14.3	7.3		17.3	7.7	
High	17.7	7.2		14.0	7.3		16.6	7.9	
SPTSS									
No	20.2	6.6	< 0.001	16.5	7.1	< 0.001	19.2	7.3	< 0.001
Avoidance	17.4	7.2		13.4	7.2		16.1	7.9	
No	20.0	6.6	< 0.001	16.3	7.1	< 0.001	18.9	7.4	< 0.001
Arousal	18.0	7.2		14.1	7.3		16.8	7.9	
No	20.1	6.5	< 0.001	16.3	6.9	< 0.001	18.8	7.3	< 0.001
Re-experience	17.8	7.3		13.9	7.5		16.7	8.0	
None	20.6	6.4	< 0.001	17.1	6.9	< 0.001	19.4	7.0	< 0.001
One SPTSS symptom	20.1	6.6		16.4	6.8		19.1	7.5	
Two SPTSS symptoms	18.7	7.0		14.5	7.3		17.6	7.7	
Three SPTSS symptoms	16.9	7.4		13.1	7.3		15.7	7.9	

*NS* Not significant

One-war ANOVA test was used in this table

Through the same software, odds ratios (ORs) and the 95% confidence intervals for the groups were calculated using the Mantel–Haenszel test. Values of less than 0.05 for the two-tailed *P* values were considered statistically significant. Bonferroni correction was used to reduce type 1 error when comparing multiple variables in Tables 1 and 2. It is calculated by *P* = α\m where α in our study is 0.05 and m is the number of hypotheses.

## Results

Overall, 2173 filled in the survey. However, 198 were excluded as they did not answer all the questions, or their replies were invalid. Another 24 participants refused to be enrolled despite their initial consent when initiating the survey. The study included 1951 responders with 527 (27.0%) being males, and 1538 (78.8%) participants being within the age range of [19–25] years.

Among responders, 19.2% (CI 95%: 17.5–21%) were likely to be well, 19.5% (CI 95%: 17.8–21.3%) had a mild mental disorder, 16% (CI 95%: 14.9–18.1%) had a moderate mental disorder, and 44.7% (CI 95%: 42.6–47.0%) had a severe mental disorder according to K10 scale. Other characteristics of the subjects are demonstrated in (Table 4).

**Table 4:** Characteristics of the subjects with characteristics of war, current medical conditions, and K10 Scale

**Characteristic**	**Frequency (*n*** **= 1951)**	**Percentage%**
**Age**		
14–18	52	2.7
19–25	1538	78.8
26–45	333	17.1
46–65	28	1.4
**Gender**		
Male	527	27.0
Female	1424	73.0
**Marital Status**		
Single	1617	83.0
Engaged	86	4.4
Married	217	11.1
Divorced	21	1.1
Widowed	7	0.4
**Consanguinity**		
No	1416	72.6
Yes third-degree relatives	300	15.4
Yes fourth-degree relatives	97	5.0
Yes but not close relatives	138	7.1
**Medical Conditions**		
Negative	1008	81.9
Asthma	63	5.1
Hypertension	23	1.9
Diabetes	6	0.5
Asthma, Hypertension	6	0.5
Diabetes, Hypertension	4	0.3
Other	121	9.8
**Being distressed from the war noises**		
No	407	20.9
Yes	1544	79.1
**Educational level**		
Primary School	27	1.4
High School	127	6.5
University or any high institute	1671	85.6
Masters or PhD	126	6.5
**Type of work**		
At a company	103	6.2
Clerk or at a restaurant	21	1.3
Education	104	6.3
Freelancer	78	4.7
Journalism	4	0.2
Law	10	0.6
Health care	234	14.2
Unemployed	1100	66.5
**SES Level**		
Lower	30	1.5
Upper Lower	483	24.8
Middle	1044	53.5
Upper	394	20.2
**Losing someone due to the war**		
No	697	35.7
Yes	1254	64.3
**Changing area of living due to war**		
No	977	50.1
Yes within the same city	429	22.0
Yes with changing the city	488	25.0
Yes I had to do both	57	2.9
**Number of times changing place of living due to war**		
No	977	50.1
Once	224	11.5
Twice	211	10.8
Thrice and more	539	27.6
**Place of origin**		
Damascus, Rif-Dimashq, and Aleppo	1045	53.6
Homs and Hama	354	18.1
Al-Jazira region	94	4.8
Southern Syria	142	7.3
Syrian coast	200	10.3
Idlib	82	4.2
Other	34	1.7
**A relative being endangered by the war**		
No	284	14.6
Yes	1667	85.4
**Characteristic (*n*** **= 1951)**	**Frequency (Percentage%)**	**CI:95%**
**Kessler psychological distress Scale (K10)**		
No	376 (19.3%)	17.5–21.0%
Mild	381 (19.5%)	17.8–21.3%
Moderate	322 (16.5%)	14.9–18.1%
Severe	872 (44.7%)	42.6–47.0%
**SPTSS items**		
Avoidance	1040 (53.3%)	51.2–55.6%
No Avoidance	910 (46.6%)	44.3–48.8%
Arousal	1257 (64.4%)	62.4–66.5%
No Arousal	693 (35.6%)	33.4–37.5%
Re-experience	1150 (58.9%)	56.9–61.2%
No Re-experience	800 (41.0%)	38.7–43.0%
Two or more	1186 (60.8%)	58.7–62.7%
Full SPTSS symptoms	720 (36.9%)	35.0–39.1%
**MSPSS Family**		
Low	402 (20.6%)	18.8–22.4%
Medium	575 (29.5%)	27.6–31.5%
High	971 (49.8%)	47.6–52.2%
**Friends**		
Low	738 (37.8%)	35.5–40.2%
Medium	666 (34.2%)	31.7–36.3%
High	546 (28.0%)	26.2–30.2%
**Significant other**		
Low	511 (26.2%)	24.3–28.3%
Medium	613 (31.4%)	29.4–33.5%
High	826 (42.4%)	40.1–44.7%
**Total MSPP**		
Low	452 (23.2%)	21.3–25.1%
Medium	830 (42.5%)	40.3–44.6%
High	669 (34.3%)	32.2–36.5%

According to SPTSS questionnaire, 53.3% (CI 95%: 51.2–55.6%) met the criteria for having symptoms of avoidance, 64.4 (CI 95%: 62.4–66.5%) for hyper-arousal, 58.9% (CI 95%: 56.9–61.2%) for re-experience, and 36.9% (CI 95%: 35.0–39.1%) for full PTSD symptoms.

### K10 results

According to Table 2, there were significantly higher K10 scores in participants who changed places of living multiple times due to war, females, participants with low SES, low educational levels, younger age groups, and participants who were distressed from war noise. When regressing the previous significant variables on K10 scores by using forward linear regression, they were all significant (*P* < 0.05). Regression model is demonstrated in (Table 5). Other variables and their correlation with K10 scores are demonstrated in (Table 2).

**Table 5:** Demonstrating forward linear regression on K10 scores, MSPSS, Avoidance, Hyper-arousal, and re-experience score with their relevant statistically significant variables

	Model	R	R^**2**^	Adjusted R^**2**^	Std. Error of the Estimate	Change Statistics					Unstandardized Coefficients β	Standardized Coefficients β	Sig.
						R^**2**^ Change	F Change	df1	df2	Sig. F Change			
**K10 score**	**Number of time changing place of living**	.125^a^	.016	.015	9.757	.016	30.728	1	1948	.000	.838	.106	.000
	**Gender**	.160^b^	.026	.025	9.709	.010	20.440	1	1947	.000	−2.098	−.095	.000
	**SES**	.191^c^	.037	.035	9.657	.011	21.896	1	1946	.000	−1.481	−.107	.000
	**Educational level**	.207^d^	.043	.041	9.629	.006	12.350	1	1945	.000	−1.539	−.067	.004
	**Age group**	.212^e^	.045	.043	9.620	.002	4.803	1	1944	.029	−1.100	−.053	.022
	**Being distressed from war noise**	.218^f^	.047	.044	9.610	.002	4.861	1	1943	.028	1.224	.051	.028
**MSPSS score**	**SES**	.151^a^	.023	.022	18.010	.023	45.328	1	1945	.000	3.865	.151	.000
**Avoidance score**	**Number of time changing place of living**	.106^a^	.011	.011	1.854	.011	22.114	1	1947	.000	.150	.100	.000
	**Educational level**	.119^b^	.014	.013	1.852	.003	5.702	1	1946	.017	−.236	−.054	.017
**Hyper-arousal score**	**Being distressed from war noise**	.126^a^	.016	.015	1.455	.016	31.549	1	1947	.000	.419	.116	.000
	**SES**	.159^b^	.025	.024	1.449	.009	18.503	1	1946	.000	−.184	−.089	.000
	**Number of time changing place of living**	.180^c^	.032	.031	1.444	.007	14.430	1	1945	.000	.093	.079	.000
	**Educational level**	.188^d^	.035	.033	1.442	.003	5.789	1	1944	.016	−.185	−.054	.016
**re-experience score**	**Being distressed from war noise**	.114^a^	.013	.012	1.610	.013	25.648	1	1947	.000	.440	.110	.000
	**Losing someone due to war**	.153^b^	.024	.023	1.601	.011	21.020	1	1946	.000	.349	.103	.000
	**Educational level**	.173^c^	.030	.029	1.596	.007	13.063	1	1945	.000	−.306	−.081	.000

**Fig. 1:**
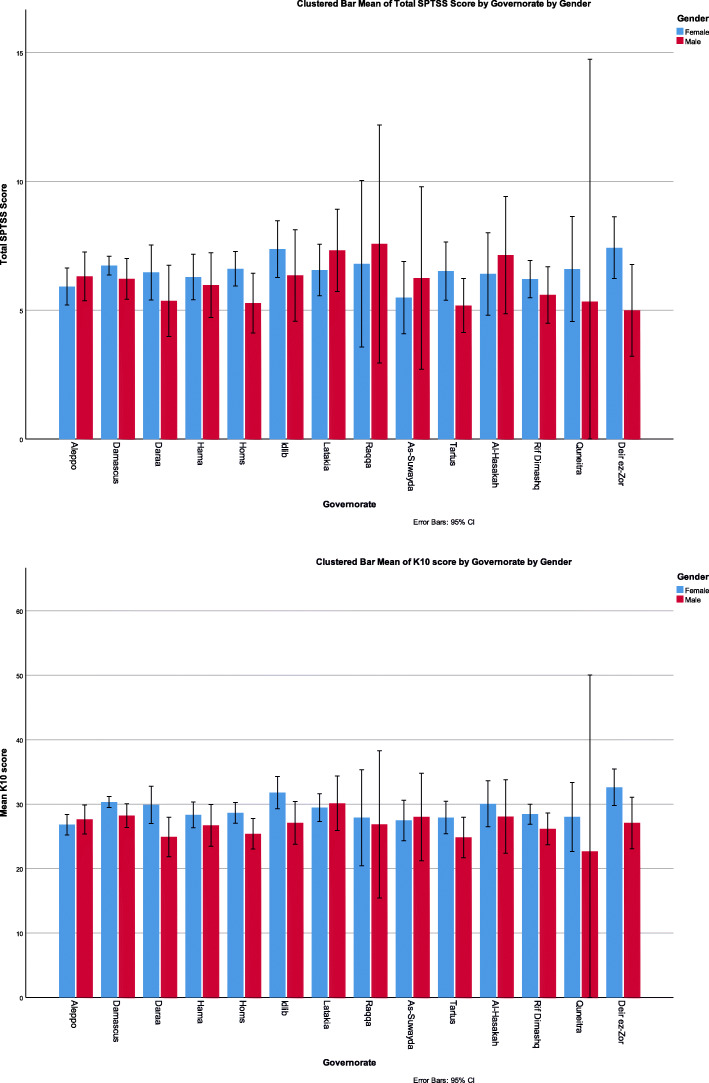
Showing governorate of current living of the subjects with their K10 scale and full SPTSS according to their gender

There was no statistically significant difference when comparing different K10 results with governorates of current living (*P* = 0.219). However, disturbance of K10 scores among governorates is demonstrated in (Fig. 1). K10 mean scores were higher by at least 3 points when having one or more chronic medical conditions when compared to not having any (*P* < 0.001, F = 7.6).

**Fig. 2:**
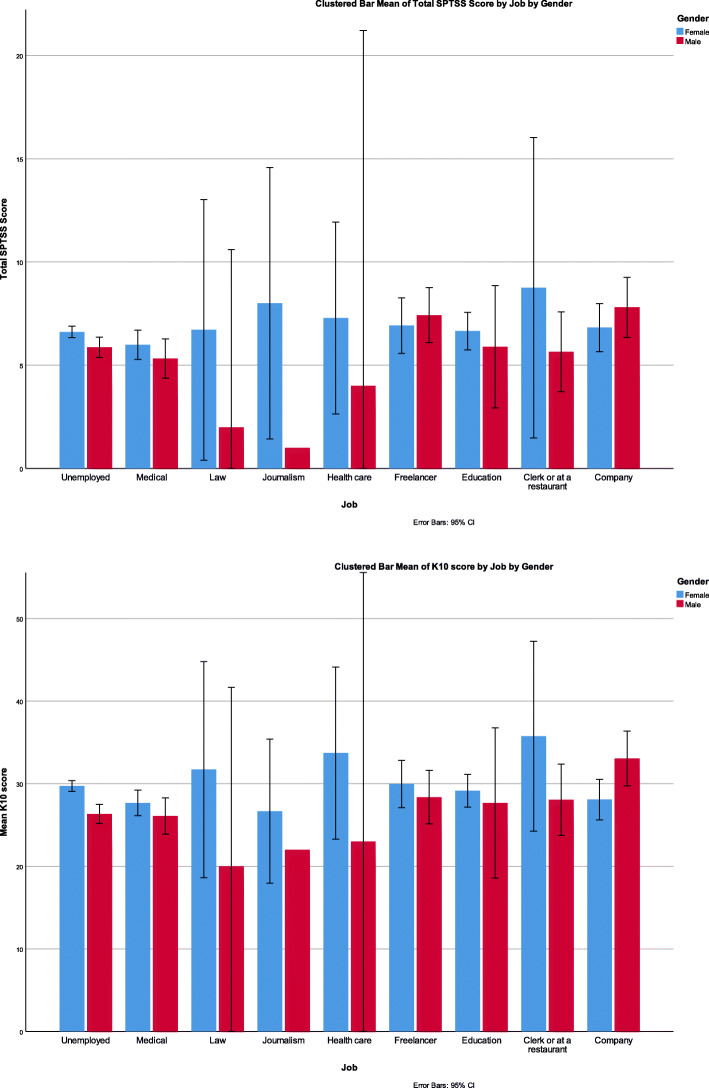
Showing SPTSS and K10 mean scores according to occupation

When using Pearson correlation, positive correlations (*P* < 0.001) were found between K10 scores and higher numbers of days being unable to work, study, or manage daily activities because of the negative feelings (*r* = 0.110), more frequent visits to a health professional because of the negative feelings (*r* = 0.110), and more frequent physical problems being attributed to the negative feelings (*r* = 0.180). K10 scores were not correlated with the type of work (*P* = 0.357) and are demonstrated in (Fig. 2).

### MSPSS results

When comparing the governorate of current living and MSPSS, we did not find a statistically significant difference *P* = 0.662. When using one-way ANOVA, high overall support was associated with fewer times of reporting physical problems being attributed to the negative feelings (*P* = 0.040). However, *P*-value was higher than 0.05 with other LM questions.

According to Table 2, significantly higher MSPSS scores were found in engaged participants, and with higher SES groups (*P* < 0.001). When regressing the previous two variables from Table 2 on MSPSS scores by using forward linear regression, only SES was found significant (*P* < 0.001) with an *R*^2^ of 2.3%. Regression model is demonstrated in (Table 5).

### SPTSS results

Comparing SPTSS clusters with each variable is demonstrated in (Table 1). SPTSS total scores were not associated with the type of work (*P* = 0.357) and they are demonstrated in (Fig. 2). When comparing the governorate of current living with having PTSD, no statistically significant difference was observed *P* = 0.681. However, disturbance of K10 scores among governorates is demonstrated in (Fig. 1). The mean SPTSS score was higher by at least 4.5 points when having one or more chronic medical conditions compared to not having any (*P* < 0.001, F = 12.6).

We used forward linear regression on avoidance scores with the statistically significant variables of avoidance, except for the variables with an asterisk in Table 1. This showed the number of times changing places of living, and education were statistically significant (*P* < 0.05). When using the same method on hyper-arousal, distress from war noise, number of times changing places of living, and education were statistically significant (*P* < 0.05). When using the same method on re-experience, distress from war noise, losing someone due to war, educational level were statistically significant (*P* < 0.05). All regression models are demonstrated in (Table 5).

### Comparisons between K10, SPTSS, and MSPSS results

When using independent t-test, the mean score of K10 was 32.9 (±23.9) in participants with avoidance symptoms, 23.9 (±8.4) in participants without avoidance symptoms, and t = − 22.69 (*P* < 0.001). The mean score of K10 was 32.1 (±9.2) in participants with hyper-arousal symptoms, 22.6 (±7.9) in participants without hyper-arousal symptoms, and t = − 22.76 (*P* < 0.001). The mean score of K10 was 32.3 (±9.2) among participants with re-experience symptoms, and 23.5 (±8.2) in participants without re-experience symptoms, and t = − 21.78 (*P* > 0.001).

When using one-way ANOVA, MSPSS scores were significantly associated with worse K10 results and SPTSS clusters which are demonstrated in **(**Table 3**)**. When using Pearson correlation, higher K10 and SPTSS symptoms scores were significantly associated with lower family, friends, and significant other support. Family support score was significantly (*P* < 0.001) correlated with K10 score (*r* = − 0.195), avoidance score (*r* = − 0.236), arousal score (*r* = − 0.195), and re-experience score (*r* = − 0.192). Friend support score was also significantly (*P* < 0.001) correlated with K10 score (*r* = − 0.171), avoidance score (*r* = − 0.257), arousal score (*r* = − 0.214), and re-experience score (*r* = − 0.192). Finally, significant other support was significantly (*P* < 0.001) correlated with K10 score (*r* = − 0.160), avoidance score (*r* = − 0.233), arousal score (*r* = − 0.177), and re-experience score (*r* = − 0.157).

### Other results

When using one-way ANOVA and independent t-test, more days were declared being unable to work, study, or manage daily life because of the negative feelings among subjects with more SPTSS symptoms (*P* = 0.061), avoidance symptoms (*P* = 0.0190), hyper-arousal symptoms (*P* = 0.034), and re-experience symptoms (*P* = 0.043). Fewer subjects declared they could work but had to cut down on what they did which was statistically insignificant (*P* > 0.05). Furthermore, more frequent visits to health professionals because of what was felt was associated with total PTSD symptoms (*P* = 0.063), avoidance symptoms (*P* > 0.05), hyper-arousal symptoms (*P* = 0.022), and re-experience symptoms (*P* = 0.022). More subjects also declared that having physical health problems being the main cause of the negative feelings was associated with more SPTSS symptoms (*P* < 0.0001), avoidance symptoms (*P* = 0.003), hyper-arousal symptoms (*P* < 0.0001), and re-experience symptoms (*P* < 0.0001).

## Discussion

### Distress, depression, and mental disorder

Our study showed that 80.7% of people in Syria scored above 20 in the K10 test, and around 60% of the population reported symptoms consistent with moderate to severe mental disorder. Changing places of living multiple times due to war, being females, having a low SES, low educational levels, younger age groups, and being distressed from war noise were associated with higher K10 scores. High K10 scores were also correlated with more frequent visits to the doctor, more days off work, and more physical problems. Over 80% of the participants were younger than 25 years of age. We did not find significant differences when comparing mental disorders among governorates and different types of jobs.

Among a typical population, 13% scored 20 or above in the K10 questionnaire. In primary care, around 25% of patients in primary care scored 20 or over in high-income countries [17, 18]. These numbers are much higher in low-income countries; in Iraq, around 60% of Syrian refugees had probable depression [25]. Another study found that 56% of the Syrian refugees at Alzatary Camp in Jordan suffered from mental distress, and 46% believed they needed mental support [26]. At least 49.7% of the refugees in Germany were screened positive for a mental disorder, with 21.7% having depression and 10.3% having major depression [27]. Whilst depression was the most common mental disorder among Syrian refugees in Sweden with the prevalence being 40.2% [28], a study on Syrian school students found that the depression rate was 32% in 2018 [29].

Several factors increase the risk of developing mental distress. It is known that the most important period for the development of the mental balance is during the adolescence. Unfortunately according to some studies, around 20% of young people in the world suffer from mental problems [30]. Many studies revealed that mental disorders prevalence is often two times higher among females than males [28, 31]. Male Syrian refugees also reported facing more traumatic events [25].

### PTSD and traumatic exposure

In this study, 36.9% of the participants had full PTSD symptoms, 60.8% had two or more positive PTSD symptoms, and only 21% did not have any PTSD symptoms. Our study showed that 49.9% had to change places of living due to war, with 27.6% having to change their place of living three times or more. Moreover, 64.3% lost someone due to war, and 85.4% had a relative or a close friend who was endangered by war. The number of times changing places of living due to war, educational level, and distress from war noise contributed the most to the high PTSD scores. We did not find significant differences in PTSD prevalence among governorates and different types of jobs.

Around 60% of Syrian students in Syria have PTSD and/or problematic anger [32]. One study in 2019 found that 61.4% of Syrian refugees met the DSM-5 symptom criteria for probable PTSD with a significant difference between males and females [25]. However, another study on Syrian refugees in Lebanon found that 27.2% had a PTSD point prevalence and 35.4% had a lifetime prevalence [33]. In Turkey, it was found that the prevalence of PTSD was 33.5% among Syrian refugees using DSM-IV-TR criteria [34]. Moreover, 34.9% of refugees in Germany had PTSD [27] compared to 29.9% in Sweden [28], and 35.1% in Syria [29].

Approximately 70% of people experience at least one traumatic incident in their life [35]. This might cause persistent avoidance and re-experiencing of the event in addition to other symptoms that reveal an emotional stimulation or a stress response [36, 37]. Around 10–40% of trauma survivors will develop PTSD [38] which is associated with a decreased quality of life [39]. Prevalence rates of PTSD are widely varied across studies due to differences in measures and periods in which the studies were conducted. Moreover, 59.1% were secreened positive for trauma exposure in Syria. Furthermore, refugees from Aleppo had higher PTSD prevalence than refugees from Homs [33]. Another study conducted in Syria on school students showed that 50.2% of participants were internally displaced [29]. Another study on Syrian refugees in Iraq showed that 98.5% of refugees had encountered at least one traumatic event, and 86.3% of them encountered at least three [25].

Other variables also took part such as age, gender, illness history, level of social support, and cultural background [40]. Besides, a study on Syrian refugees in Turkey found that experiencing two or more traumatic events significantly increased the risk of PTSD, and the ratio of females having PTSD was four times higher than males [34]. Numerous studies about gender differences showed that males are less likely to develop PTSD after traumatic events, and therefore the prevalence of PTSD among women will be higher. In contrast, a study in Lebanon showed no significant differences in PTSD and depression rates between male and female university students who faced war-related trauma [41].

Age is considered a significant risk factor for developing PTSD; a meta-analysis of 29 studies on trauma-exposed adults revealed that exposure to a traumatic event at a younger age was an important risk factor for PTSD [42].

### Social support

Although only 23.2% of our sample had a low total support, the high prevalence of PTSD, and mental disorders suggest other factors being involved besides low support levels as (*r* < 0.3) which is weak. SES was the most contributing factor to social support.

The literature indicates that social support was a preventive factor for the development of PTSD for men and women. Furthermore, the incidence of post-traumatic stress increased in those with a low social support. For many who have experienced trauma in their lives, social support was a preventive factor for the development of PTSD [43]. This confirms our finding of the negative association between MSPSS scores, and SPTSS and K10 scores (*P* < 0.0001).

However, after a long period of exposure to trauma, the impact of social support as a protective factor may be mitigated [44]. A study at Alzatary Camp in Jordan found that 66.7% of refugees staying at the camp reported a great need for mental support [26].

### Outcomes of the psychological burden

Being fearful, easily angered, nervous, having difficulty sleeping or staying asleep, absence of hope for the future, and spells of terror or panic were some of the characteristics that the Syrian refugees experienced at Alzatary Camp in Jordan [26]. Similarly, 31.8% of refugees in Sweden, and 29.5% of Syrian students had anxiety [28, 29]. Other studies in Syria found that dental and genitival health were associated with PTSD and mental disorders [45, 46]. Another study found that around 50% of the population had allergic rhinitis which could be from the direct or indirect effects of war or the unique environment [47]. A similar study found a high prevalence of laryngopharyngeal reflux which is also related to war variables [48]. Smoking is also common among the Syrian population, mainly social shisha smoking which could be to get away from the daily stress. Shisha smoking is mainly common among university students who represent most of our study [49].

Moreover, war has affected university students [50], and prevented research from being properly conducted due to a shortage of resources [51]. Many studies lacked proper funding which ultimately generated limited data. There are many crucial medical investigations and procedures not conducted in most studies in Syria, and delayed treatment can occur due to the financial hurdles which can dramatically affect patients’ care [12–16]. This reflects some of the negative outcomes that Syrians have endured, especially in those who were mostly affected by the war.

The stigma of mental health in Syria is very common, and only a few practicing psychiatrists and psychotherapists exist. As social support was only weakly but significantly correlated with lower K10 and SPTSS scores (*r* < 0.3 with *P* < 0.001), other measurements are required to boost mental health in the society. National-wide programs are needed to increase awareness, and humanitarian assistance is required to benefit from international experts in mental health. Financial assistance is also needed as the deteriorating financial situation is a strong contributing factor to the suffering.

### Limitations

Most online surveys in Syria tend to include the young population and females more than males as this population tends to be members in social media groups more often. In contrast, older generations exist mainly in family’s and close friends’ groups, and they are disinterested in filling in surveys that are not directly sent from a person they know, or they simply do not know how to fill them in. This pattern is seen in multiple online studies from Syria. This might have affected the results as the young might react differently compared to the elder. Self-reported symptoms also tend to overestimate the true prevalence of mental symptoms. Besides, the nature of the method – self-reported questionnaires – solicits responses which may vary depending on the participant’s feelings at the time.

Although K10 is a good screening method to detect recent anxiety and depressive symptoms, it is not an appropriate alternative for medical consultation. However, after clinical diagnosis, K10 can be used for assessment as scores that remain above 24 are indicative to the needs for a referral to a specialist [17, 18]. Symptoms associated with PTSD can also be seen in the normal phase of dealing with stress which the Syrian population has been experiencing since 2011, with no periods that allowed for mental healing or stability.

Furthermore, mental illness rates can be associated with factors that have not been addressed in our study. For example, studies among war-affected displaced populations showed that the number of traumatic events was associated with increased mental illness rates as previously discussed; we could not, however, determine the exact event(s) that the population had faced. Moreover, geographical characteristics appear to influence the psychological wellbeing of displaced populations. Most studies showed that severe mental disorders were more common in cities compared to rural areas [25, 52]. However, one study on Syrian refugees found this difference was only with PTSD, not with depression symptoms [25]. Our study could not determine the exact place of living, whether it was urban or rural. We could only determine the governorate of origin since responders might have been displaced several times which made it difficult to determine this factor.

This study did not consider the mental background of participants, which could have aggravated the symptoms of PTSD or biased the questionnaire. This study was online which made it difficult to determine the population at risk. Moreover, responders who had an internet connection and were willing to do the questionnaires are probably in a better mental condition than those who are truly severely affected, and do not have internet connection or the will to do the survey. Finally, most of the responders were university students with potentially higher SES than the normal population. For all the previous reasons, this study might have underestimated the true prevalence of distress amongst the general population.

SES could not be accurately determined since asking about the salary is inappropriate in the Syrian culture. There is a huge difference in living costs in Syria, where people can live of a lower income compared to other countries in the region. SPTSS is based on DSM-IV, not V. However, it can resemble ICD-11.

## Conclusion

The Syrian conflict has caused severe mental distress in the Syrian society. Efforts and interventions to improve the psychological wellbeing of the Syrian population are needed to ensure the people are prepared for the reconstruction of their country when the situation improves. The results of this study reflect the underlying disaster that made people severely mentally impaired. The social support had a relative weak effect, meaning that resources should be re-allocated to other aspects of care. Syrian people have serious concerns about financial and psychiatric aspects of their lives and require various measures to ameliorate their situation. These results also emphasises on the importance of security either economically or in terms of personal safety which are more important in the long term than support from family, friends, and the significant other as higher mental distress is seen in those who remained in Syria compared to refugees in camps.

This study suggests that internally displaced people, and even those who were not displaced in the conflict, experienced more severe mental disorder than the Syrian refugees in most countries. The number of times changing places of living due to war and being distressed from war noise have contributed the most to the high PTSD, anxiety, and depression burden. More than 60% of the population suffered from PTSD and severe mental disorders. Having a high educational background was associated with less severe mental disorders and PTSD. Moreover, a higher number of times changing places of living due to war, a lower educational level, and being distressed from war noise were associated the most with sever PTSD and mental distress. No significant differences in mental disorders, or PTSD were noted among participants from different governorates or with different job types. Many other variables have contributed to these findings altogether which indicate the need for addressing multiple issues. Females and young participants suffered more on the psychiatric aspect.

## Data Availability

The data can be made available upon reasonable request.

## Abbreviations

ANOVA: Analysis of variance
CI: Confidence Interval
DSM: Diagnostic and statistical manual of mental disorders
ICD: International Classification of Disease
K10: Kessler
MSPSS: Multidimensional Scale of Perceived Social Support
OR: Odds ratios
PTSD: Post-traumatic stress disorder
SES: Socioeconomic status
SPSS: Statistical Package for the Social Sciences
SPTSS: Screen for Posttraumatic Stress Symptoms
